# SMARC-B1 deficient sinonasal carcinoma metastasis to the brain with next generation sequencing data: a case report of perineural invasion progressing to leptomeningeal invasion

**DOI:** 10.1186/s12885-019-6043-0

**Published:** 2019-08-22

**Authors:** Horacio Gomez-Acevedo, John D. Patterson, Sehrish Sardar, Murat Gokden, Bhaskar C. Das, David W. Ussery, Analiz Rodriguez

**Affiliations:** 1 Department of Biomedical Informatics, University of Arkansas for Medical Science, Little Rock, AR 72205 USA; 2 Department of Neurosurgery, University of Arkansas for Medical Sciences, Little Rock, AR 72205 USA; 3 Division of Neuropathology, Department of Pathology, University of Arkansas for Medical Sciences, Little Rock, AR 72205 USA; 4 Department of Pharmacological Sciences, Icahn School of Medicine at Mount Sinai, New York, NY 10029 USA

**Keywords:** SMARCB1 deficient sinonasal carcinoma, Perineural invasion, Head and neck carcinoma, Leptomeningeal carcinomatosis, Next generation sequencing

## Abstract

**Background:**

SMARCB1-deficient sinonasal carcinoma (SDSC) is an aggressive subtype of head and neck cancers that has a poor prognosis despite multimodal therapy. We present a unique case with next generation sequencing data of a patient who had SDSC with perineural invasion to the trigeminal nerve that progressed to a brain metastasis and eventually leptomeningeal spread.

**Case presentation:**

A 42 year old female presented with facial pain and had resection of a tumor along the V2 division of the trigeminal nerve on the right. She underwent adjuvant stereotactic radiation. She developed further neurological symptoms and imaging demonstrated the tumor had infiltrated into the cavernous sinus as well as intradurally. She had surgical resection for removal of her brain metastasis and decompression of the cavernous sinus. Following her second surgery, she had adjuvant radiation and chemotherapy. Several months later she had quadriparesis and imaging was consistent with leptomeningeal spread. She underwent palliative radiation and ultimately transitioned quickly to comfort care and expired. Overall survival from time of diagnosis was 13 months. Next generation sequencing was carried out on her primary tumor and brain metastasis. The brain metastatic tissue had an increased tumor mutational burden in comparison to the primary.

**Conclusions:**

This is the first report of SDSC with perineural invasion progressing to leptomeningeal carcinomatosis. Continued next generation sequencing of the primary and metastatic tissue by clinicians is encouraged toprovide further insights into metastatic progression of rare solid tumors.

**Electronic supplementary material:**

The online version of this article (10.1186/s12885-019-6043-0) contains supplementary material, which is available to authorized users.

## Background

Sinonasal carcinomas have recently gained increased interest for their histogenetically diverse characteristics brought to light by advances in molecular genetics [1]. These advanced techniques in molecular genetics have diversified the classification of the highly aggressive sinonasal undifferentiated carcinomas into more precise classifications based on their genetic alterations and biologic features [2]. Among these new tumor variants, such as NUT-rearranged carcinoma [3, 4], HPV-related adenoid cystic-like carcinoma [5, 6], and adamantinoma-like Ewing sarcoma [7], SMARCB1-deficient sinonasal carcinoma (SDSC) stands out given its aggressiveness in the face of multimodal therapy.

SDSC is a rare, often fatal tumor characterized by distinct inactivating alternations of the tumor suppressor gene SMARCB1 located on chromosome 22q11.2, basaloid/rhabdoid differentiation, and histologic loss of INI1 expression [8]. With fewer than 80 cases of these distinct tumors being reported in the literature, much is left to be known about the morphologic and genetic features, as well as other tumor characteristics such as metastasis and treatment efficacy. Many of these cases are summarized by recent case series done by Agaimy et al. [9] and Kakkar et al. [10] and demonstrate that while epidural metastasis of SDSCs is not uncommon, intradural metastasis was reported in only 3/52, while intradural metastasis with concurrent perineural spread in only 1/52 cases. In this report, we describe and discuss the clinical course of an aggressive case of SDSC with perineural involvement that recurred following radiation therapy, metastasized intradurally to the central nervous system, and subsequently developed into leptomeningeal spread. This article also features the first report on genetic sequencing data for a SMARB1 deficient carcinoma brain metastasis.

## Case presentation

**Fig. 1:**
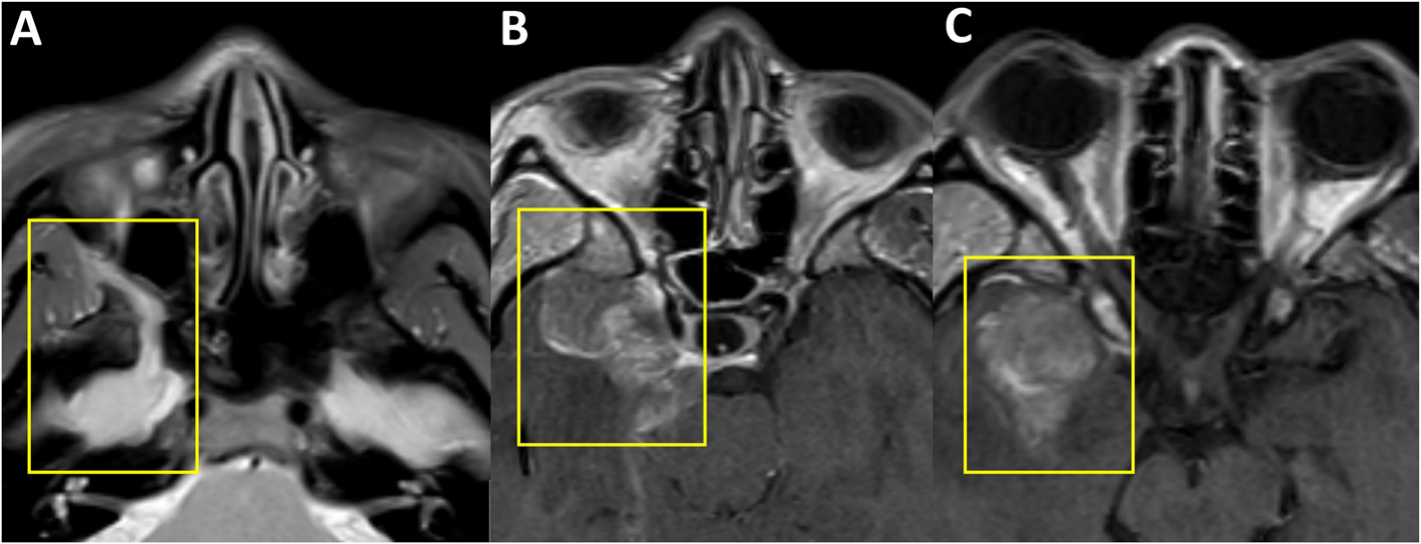
Radiographic Images of the Primary Sinonasal Carcinoma with perineural extension. Magnetic resonance images in the axial planes are presented. Preoperative images demonstrate a right sided mass along the trigeminal nerve (**a**). Following adjuvant radiation, the patient had progression with extension of tumor into the cavernous sinus (**b**) and an associated intradural metastasis (**c**). Yellow boxes highlight the tumor region of interest

**Fig. 2:**
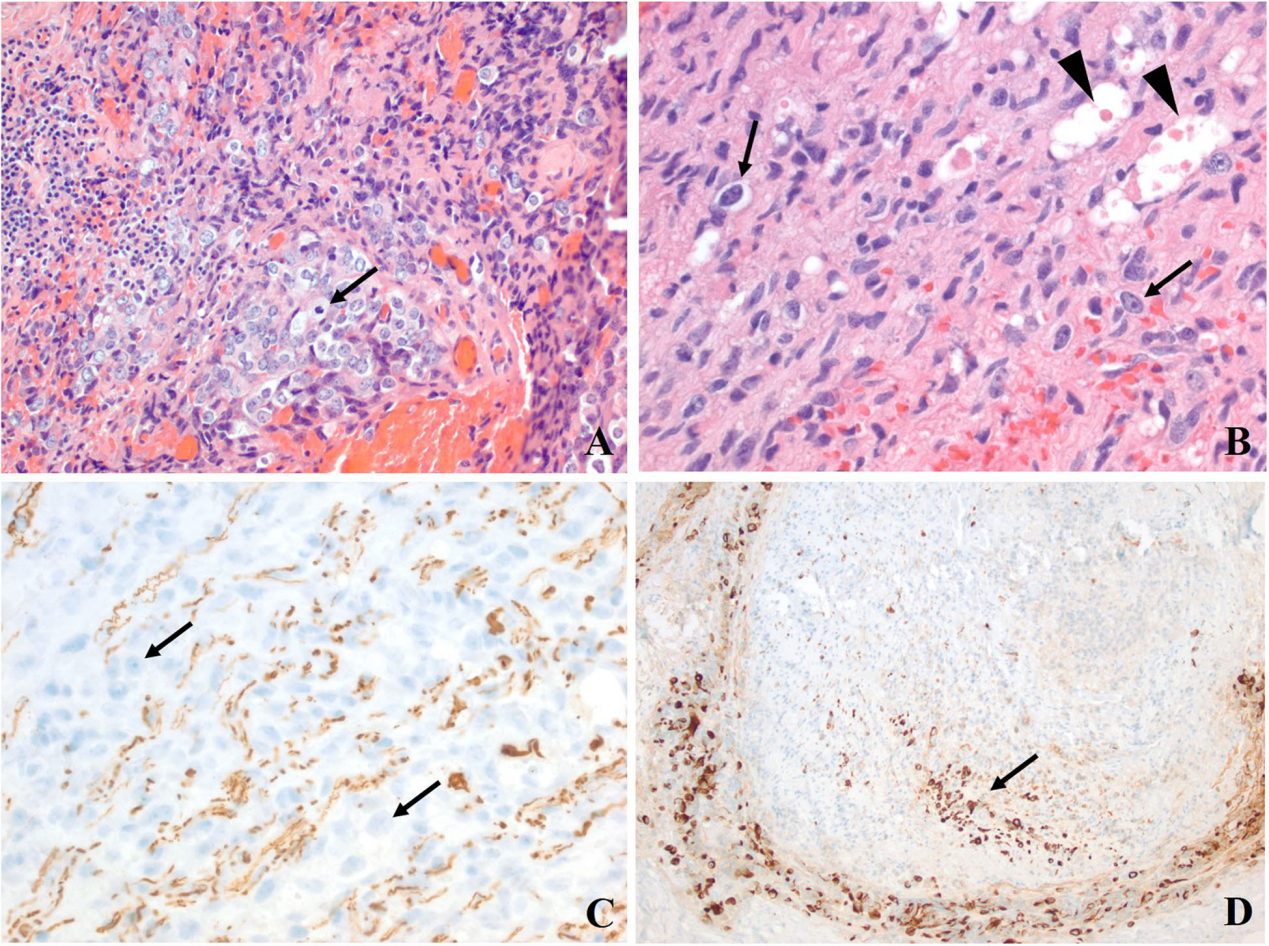
Histologic findings of initial biopsy specimen. **a**. A poorly-differentiated neoplasm with large epithelioid cells and mitotic activity (arrow) is seen. **b**. The neoplasm (bottom right) infiltrates the nerve fascicles (upper left). Many large, atypical cells (arrows) crowding the fascicle, which is represented by residual spindled Schwann cells, and several degenerating axons with myelin ovoids (arrowheads).**c**. Neurofilament protein immunohistochemistry highlights the axons that are widely separated from each other by infiltrating neoplastic cells. **d**. Cytokeratin shows the neoplasm to be a carcinoma encircling the nerve fascicle, with a group of infiltrating cells (arrow) within it. (**a** and **b**: Hematoxylin and eosin; **c** and **d**: Immunohistochemistry. Original magnifications: **a** and **d**: 200x; **b** and **c**: 400x)

A 42 year old female presented with a right sided headache with associated right sided facial pain. A Computed Tomography (CT) scan showed a mass on the V2 division of the trigeminal nerve (Fig. 1a). A maxillectomy was performed for resection and pathology returned a diagnosis of a SMARCB1-Deficient Sinonasal Carcinoma (Fig. 2). Several weeks later the patient received adjuvant Gamma Knife Stereotactic Radiosurgery (GKSRS) to the remaining perineural mass at 18 Gy to the 50% Isodose line.

**Fig. 3:**
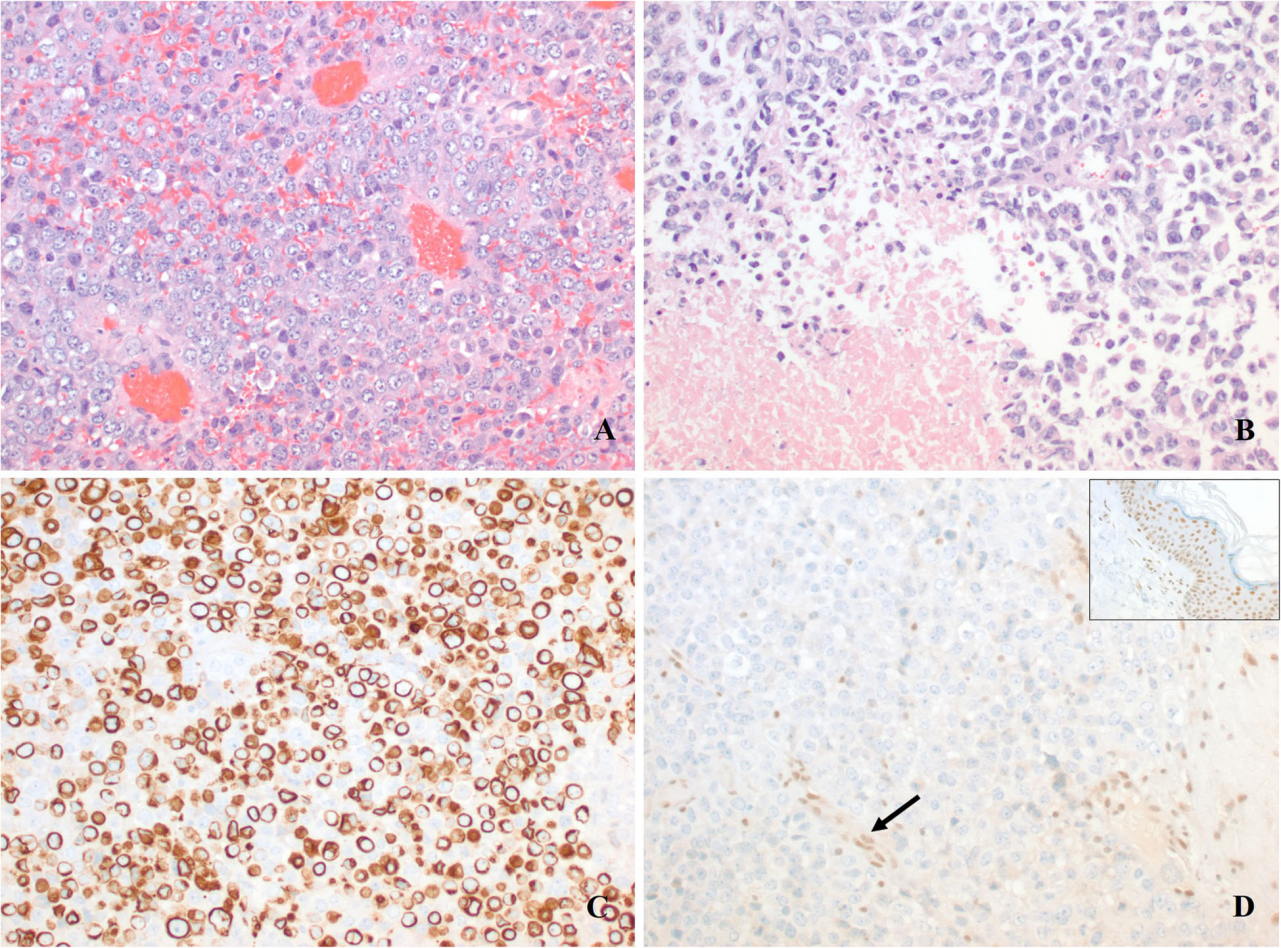
Histologic findings of intracranial resection specimen. **a**. Sheets of highly atypical cells with open chromatin and prominent nucleoli, resembling nasopharyngeal undifferentiated carcinoma. **b**. Areas of necrosis and scattered cells with rhabdoid features are also seen. **c**. Many cells are positive for low-molecular weight cytokeratin (CK 8/18). **d**. Loss of nuclear INI-1 expression in the neoplastic cells, while it is retained in the endothelial cells (arrow). Inset: INI-1 control stain. (**a** and **b**: Hematoxylin and eosin; **c** and **d**: Immunohistochemistry. Original magnifications: **a**-**d**: 200 x)

**Fig. 4:**
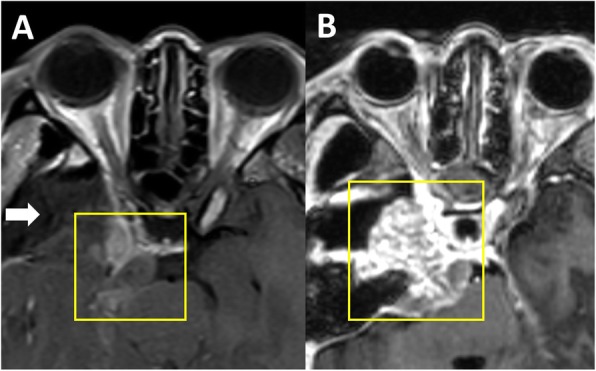
Radiographic Images of Sinonasal Carcinoma following intracranial surgical resection. Magnetic resonance images in the axial planes are presented. There was a gross total resection of the brain metastasis (as denoted by the white arrow) but progression of tumor in the prepontine cistern ventral to the brain stem (**a**). Two weeks later, planning imaging demonstrates further progression (**b**)

**Fig. 5:**
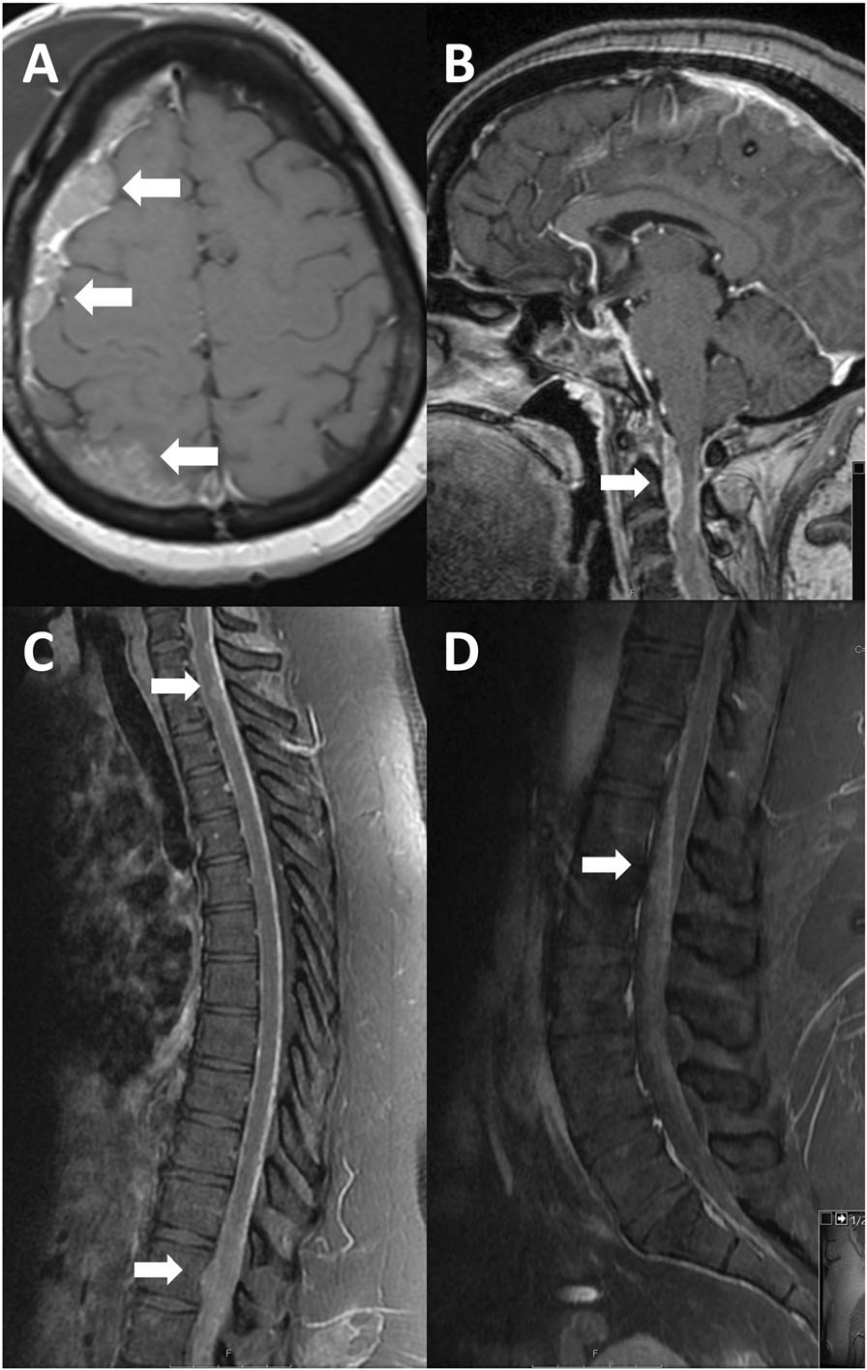
Radiographic images of leptomeningeal progression. Magnetic resonance images of the brain and spine are presented in the axial and sagittal planes, respectively. The tumor metastasized to the subdural space along on the right convexity (**a**) and the ventral cervical spine (**b**). Multiple areas of enhancement were present in the thoracic spine (**c**) and along the nerve roots of the cauda equina (**d**)

A few months after the GKSRS, the patient developed a right cranial nerve 6 palsy and severe facial pain refractory to medications. Interval imaging revealed an increase in the mass with extension along the trigeminal nerve into the cavernous sinus (Fig. 1b) and an intradural component in the temporal lobe (Fig. 1c). To resect this tumor a right sided craniotomy was performed and the mass was confirmed to be consistent with undifferentiated carcinoma (Fig. 3). For treatment of her facial pain decompression of the superior orbital fissure, foramen rotundum, foramen ovale was also performed. Postoperative imaging demonstrated that components of the tumor also extended into the right prepontine cistern (Fig. 4a). Two weeks after the craniotomy, further GKSRS was performed on the mass. Another magnetic resonance image (MRI) performed for treatment planning demonstrated further interval growth in the mass during this short interval time (Fig. 4b). Given the rapid progression of disease, palliative chemotherapy was initiated with a Cisplatin/ Etoposide regimen every 3 weeks. She reported improvement in her pain. The patient developed further significant weakness impairing her ability to ambulate approximately 3 months from her craniotomy. A MRI demonstrated leptomeningeal metastasis throughout the right cerebral convexity (Fig. 5a), extending to the upper cervical spinal cord causing compression (Fig. 5b). There was also imaging findings consistent with leptomeningeal carcinomatosis in the thoracic and lumbar spine (Fig. 5c-d). The previously radiated tumor at the skull base was largely unchanged but there was mass effect on pons. Radiation therapy was started for the spinal cord lesion. The patient became quadraparetic in all four extremities and was hospitalized. Palliative radiation therapy to the cervical and thoracic spine lesions were continued until the patient stated she would like to discontinue due to severe pain and worsening weakness. The patient became comatose and exhibited clinical signs concerning for herniation. Radiographic imaging demonstrated increase in size of subdural metastasis and mass effect causing midline shift of over 1 cm. The patient expired shortly after this radiographic study with an overall survival of 13 months.

### Experimental details

DNA and RNA sequencing were performed on two patient’s tumor specimens, one from right temporal lobe of the brain, and the other from the primary sinonasal tumor, using the xT Laboratory Developed Test at Tempus’ Clinical Laboratory Improvement Amendments/College of American Pathologists-accredited laboratory in Chicago, IL. Tumor DNA was extracted from tumor tissue sections with tumor cellularity higher than 20% and proteinase K digested. Total nucleic acid extraction is performed with a Chemagic360 instrument using a source-specific magnetic bead protocol. Total nucleic acid is utilized for DNA library construction, while RNA is further purified by DNaseI digestion and magnetic bead purification. The nucleic acid is quantified by a Quant-iT picogreen dsDNA reagent Kit or Quant-iT Ribogreen RNA Kit (Life Technologies), and quality is confirmed using a LabChip GX Touch HT Genomic DNA Reagent Kit or LabChip RNA High HT Pico Sensitivity Reagent Kit (PerkinElmer).

For the DNA library construction, one hundred nanograms of DNA for each tumor and normal sample was mechanically sheared to an average size of 200 base pairs using a Covaris ultrasonicator. The libraries were prepared using the KAPA Hyper Prep Kit. Briefly, DNA underwent enzymatic end-repair and A-tailing, followed by adapter ligation, bead-based size selection, and PCR. After library preparation, each sample was hybridized to a custom designed probe set. Recovery and washing of captured targets was performed using the SeqCap hybridization and wash kit. The captured DNA targets were amplified using the KAPA HiFi HotStart ReadyMix. The amplified target-captured libraries were sequenced on an Illumina HiSeq 4000 System utilizing patterned flow cell technology.

Generated reads were aligned to the human reference genome (hg19) using BWA aligner [11], and subsequent analysis to find somatic single nucleotide variants was carried out with FreeBayes. Somatic variants were then compared with The Cancer Genome Atlas (TCGA) reported mutations and post transcriptional modifications using ActiveDriverDB database [12]. Summary of the findings was depicted using circos [13].

RNA Library construction. One hundred nanograms of RNA per tumor sample was fragmented with heat in the presence of magnesium to an average size of 200 base pairs. The RNA then underwent first strand cDNA synthesis using random primers, followed by combined second strand synthesis and A-tailing, adapter ligation, bead-based cleanup, and library amplification. After library preparation, samples were hybridized with the IDT xGEN Exome Research Panel. Target recovery was performed using Streptavidin-coated beads, followed by amplification using the KAPA HiFi Library Amplification Kit. The RNA libraries were sequenced to obtain approximately 65 million reads on an Illumina HiSeq 4000 System utilizing patterned flow cell technology.

From RNA sequencing variant calling, the best practices published by the Broad Institute were followed. Namely, after a double pass alignment step with Star aligner [14] to the human genome hg19, reads were realigned, bases were recalibrated, and variant calls were performed with GATK [15] with specific parameters for RNAseq. Estimation of transcripts read coverage was performed with Subread [16], and log fold changes were performed to contrast brain against sinonasal samples for counts of raw reads.

### DNA single nucleotide variants obtained by next generation sequencing

**Fig. 6:**
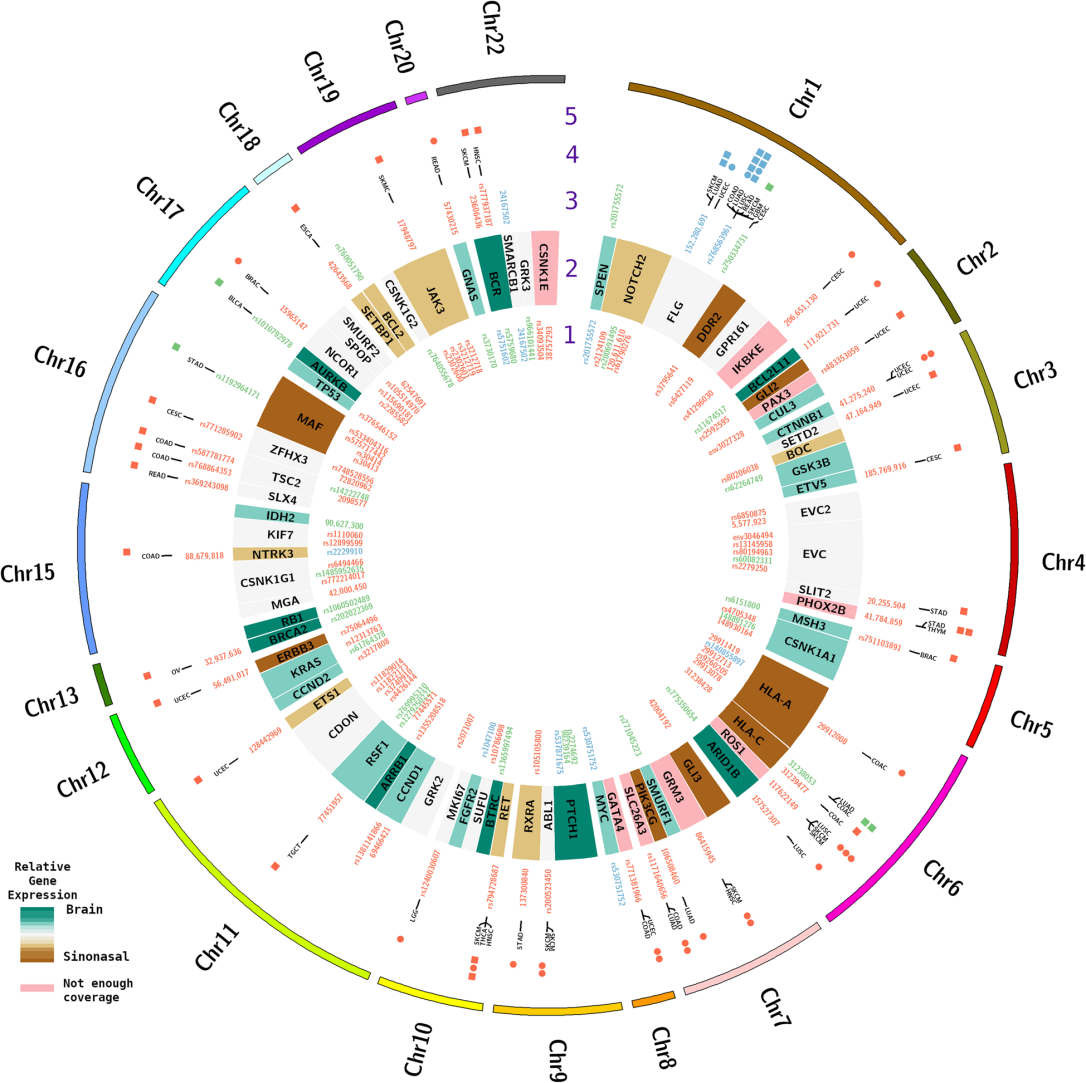
Summary of the variants on DNA and RNA and gene expression. Text in red represent findings on the sinonasal sample, green represent the brain sample and blue text represent calls present on both samples. Circular bands contain the following information: 1) SNP id or location of the variant on the RNA. 2) Gene name and relative expression levels. 3) SNP id or location of the DNA variant. 4) Matching TCGA cancer type. 5) Common variant sharing the same location (circles) or on close proximity to the variant called (squares)

The patient’s primary tumor and brain metastasis underwent next generation sequencing. We found over 500 variant calls on both samples that were screened and selected based on their mapping quality and coverage (>20x). Due to the rarity of this malignancy, we overlapped those selected variants to the mutations reported on The Cancer Genome Atlas (TCGA) using ActiveDriverDB database. We reported calls that were up to three amino acids away from the variant location and were implicated in changes in protein signaling or posttranslational modification in any malignancy (Fig. 6).

Three mutations matched TCGA cases of head and neck squamous cell carcinoma (HNSC). Namely, glutamate metabotropic receptor 3 (GRM3 D279E), ret. proto-oncogene (RET T946A), and BCR (BCR K724 N), but those mutations only appeared on the sinonasal sample. We also identified a mutation on the marker of proliferation Ki 67 (MIK67 A1218T) found on brain lower grade glioma only on the sinonasal sample. Additional file 1 contains a list of all mutations identified in both the primary sinonasal tumor and the brain metastasis.

Common variants overlapping TCGA mutations on the sinonasal and brain samples were found primarily on the filaggrin (FLG) gene, notably a missense mutation on FLG R1469H for lung squamous cell carcinoma, and another on FLG R1469L for colon adenocarcinoma. Moreover, other cancer types shown mutations close by the SNP rs768563961: skin cutaneous melanoma, lung adenocarcinoma, rectum adenocarcinoma and glioblastoma multiform. Table 1 outlines the DNA mutations identified in both samples (Table 1).

**Table 1:** DNA mutations conserved between primary tumor and brain metastasis

Chromosome	Coordinates (hg19)	Gene	Substitution	SNP	Mutation ID (TCGA)
Chr1	152,280,691	FLG	INDEL	NA	c4947f1a-f736–5156-bb47-fb99afc05eca
Chr1	152,280,691	FLG	INDEL	NA	e0e7142f-85 fc-546c-ba41-8ba6abbabbd5
Chr1	152,281,621	FLG	INDEL	NA	003298fb-bf3f-5a8d-9562-8ee223da44e3
Chr1	152,282,957	FLG	DELETION	rs767528401	3c313d6f-afb4-5db9-a879-5faf31d928a5
Chr1	152,282,957	FLG	G*/A	rs768563961	1492d3c9-6b26-50da-ac39-d86f864d9f55
Chr1	152,282,957	FLG	G*/A	rs768563961	2a7c7bad-38e4–5442-b468-70cf216611b4
Chr1	152,282,957	FLG	G*/A	rs768563961	3c298b96-29a0–5071-b876-80bcf4316002
Chr1	152,282,957	FLG	G*/A	rs768563961	43493c3f-a06d-5ef8-b014-8861a0ccfbbe
Chr1	152,282,957	FLG	G*/A	rs768563961	63aa19e0-70da-5930-a52f-c7d5a99959d2
Chr1	152,282,957	FLG	G*/A	rs768563961	8ca1a076-e066-5df3-83f2-34eba8302361
Chr1	152,282,957	FLG	G*/A	rs768563961	d53f9e7a-1176-51f1-84cf-c3db19877867

*hg 19 Homo sapiens* (human) genome assembly GRCh37, *SNP* single nucleotide polymorphism, *Indel* insertion and deletion of nucleotides, *A* adenine, *G* guanine, *indicates an exchange for that nucleotide

Brain variants overlapping TCGA mutations were considerably less than the sinonasal counterpart. There was one variant on major histocompatibility complex I gene HLA-C with one case of lung adenocarcinoma (HLA-C G276 V) and another mutation found on colon adenocarcinoma (HLA-C G276R). Another variant was found on proto-oncogene MAF (rs 1,192,964,171) close to a reported mutation on stomach adenocarcinoma (MAF V286A). Finally, on the Aurora kinase gene AURKB (rs101079278) a close mutation was reported on bladder urothelial carcinoma (AURKB S37Y).

A variant found on the apoptotic activator BCL2L11 (rs762818079) in a case of uterine corpus endometrial carcinoma shows post transcriptional modifications of the binding sequence motif for the kinases AKT1, MAPK10, MAPK8, and MAPK9.

### RNA single nucleotide variants and RNAseq expression

Gene expression data demonstrated that ten genes (i.e. SMARCB1, SMURF1, SMURF2, SPEN, SPOP, SUFU, TP53, TSC2, and ZFHX3) had at least a one log fold change in decreased expression when comparing the brain sample to the primary sample. All other genes were all less than one log fold change in expression (Additional file 2 contains the quantitative gene expression differences between samples). We extended our search for variants to RNA, focusing on genes that have been reported relevant for sinonasal carcinomas or to head and neck carcinogenesis (e.g., Sonic Hedgehog-signaling pathway). More specifically, from Dogan et al., we investigated for possible variants on RNA or DNA and relative gene expression of genes with at least 10% of mutation frequency on their cohort, and also on genes members of the KEGG Sonic Hedgehog pathway [17]. We identified a stop signal K305* on both samples for the SMARCB1 gene that was present at the RNA and DNA levels. This persistent variant may explain the constitutional inactivation of SMARCB1 present in the patient.

In addition to the previous findings on GRM 3, we saw negligible RNA levels on both samples suggesting a tumor progression similar to the apoptosis suppression observed in myeloma cell lines [18], also mutations on GRM3 have been shown to activate MEK promoting migration and proliferation in melanoma [19]. Therefore, the strong downregulation of the G protein coupled receptor GRM3 might be a relevant player on the first stages of the sinonasal carcinomas. A summary of the RNA mutations that were the same between the primary and metastatic sample are provided in Table 2. Additional file 3 contains a list of all RNA mutational variants identified in each sample.

**Table 2:** RNA Mutations conserved between primary tumor and brain metastasis

Chromosome	Coordinates (hg19)	Gene	Substitution	SNP
Chr1	16,258,505	SPEN	G*/A	rs201755572
Chr6	29,911,457	HLA-A	G*/GT	rs140855897
Chr8	128,750,408	MYC	T*/C	rs530751752
Chr9	98,240,342	PTCH1	G*/T	rs537871675
Chr10	123,298,158	FGFR2	T*/C	rs1047100
Chr15	88,576,185	NTRK3	G*/C	rs2229910
Chr22	23,523,630	BCR	C*/A	rs5751602

*Chr* chromosome, *hg 19 Homo sapiens* (human) genome assembly GRCh37, *A* adenine; *G*: guanine, *C* cytosine, *T* thymine * indicates exchange of that nucleotide

Traditionally, the Sonic Hedgehog pathway has been studied in terms of embryonic development but its presence in several malignancies suggests that either directly or indirectly this pathway stimulate the proliferation of cancer cells [20, 21]. Reception and transduction of hedgehog signaling is facilitated by a primary cilum structure to carry on the functions of this pathway [22, 23]. On the sinonasal tissue, we observed a low RNA expression of the trans membrane receptor Patched1 but high expression of the transcription factors GLI2 and GLI3. Also, the comparable between the samples of RNA-expression levels of the core regulators of the GLI proteins and kinesin family member 7 suggest that there is no suppression of GLI2 or GLI3 on either stages of cancer. Taken together, these findings suggest that the strong hyperactivity of the hedgehog signaling on the sinonasal tissue is impaired after metastasis to the brain. We also observed that the hormone inducible transcriptional repressor SPEN shows a common variant (rs201755572) at the RNA-level but only on the DNA of the brain sample, and on the latter sample its RNA expression is elevated. SPEN has been shown to promote primary cilia formation and cell migration on breast cancer [24]. Thus, it may be possible that SPEN induces cell migration on the brain, but further studies are needed given this is the only case reported in the literature of a SMARCB1 brain metastasis with genetic sequencing data.

It has been recently reported frequent mutations on the isocitrate dehydrogenases IDH2 [17, 25]. However, we were only able to identify a 3’UTR variant on the brain sample but none on the amino acid 172. We also investigated other alterations on genes that Dogan et al.1 reported as having a mutation frequency larger than 10% on their cohort [17]. Among those genes, we found the sinonasal sample had RNA variants on TP53 (rs376546152), KRAS (rs12313763), and a common variant from DNA and RNA on the oncogene MYC (rs530751752). It is worth mentioning that the brain sample had slightly higher RNA expression on IDH2, TP53, KRAS, and MYC, and considerably higher expression of the tumor suppressor gene RB1 suggesting more active tumorogenesis in the brain sample (Additional file 2). In addition, the tumor mutational burden (i.e. number of non-synonymous mutations per megabase of exonic DNA) of the brain sample was also higher than the primary sinonasal tumor (8.8 vs 3.3).

Other molecular events observed in our analysis were the high expression in the brain sample of BCR, together with a loss of heterozygosity (LOH) on the SNP rs5751602 at the RNA level. Another place that shows LOH is at rs140855897 (deletion in the brain specimen) in the major histocompatibility complex I gene HLA-A. Also, the immune system genes HLA-A and HLA-C were both highly upregulated on the sinonasal sample. Interestingly, in both samples immunohistochemistry demonstrated less than 1% PD-L1 expression in both tumor cells and tumor associated immune cells.

## Discussion and conclusions

We report a rare case of a patient with SMARC-B1 deficient tumor with perineural spread that progressed to develop into an intradural metastasis. Following treatment failure, the patient eventually developed leptomeningeal spread. Previous studies have reported the presence of brain metastasis from this tumor in 3 patients but only one patient has been previously reported as having concordant perineural spread and intradural disease, however no sequencing data was reported [10, 17]. The percentage of patients with head and neck squamous cell carcinoma that develop perineural spread varies from 14 to 63% dependent on the cohort [26]. In perineural spread, tumor cells disseminate contiguously within the perineural space into cranial nerves and eventually reach the brainstem. Our patient went on to develop leptomeningeal spread, which occurs in 3–5% of all cancer patients [27]. A recent study of 120 patients with advanced sinonasal carcinoma of various histologies demonstrated that isolated leptomeningeal progression was the most common site of isolated distant metastatic progression in 9/20 patients [28]. Mechanisms of leptomeningeal spread continue to be poorly understood. Both perineural and leptomeningeal spread are unique forms of metastasis that portend a poor prognosis [29].

To our knowledge, this is the first report that sequencing data has been reported for the primary SMARCB1 tumor with perineural spread and a matched brain metastasis. Previous whole exome sequencing of primary tumors and their accompanying brain metastasis demonstrated the potential to identify actionable alterations [30]. With the advent of targeted therapy for multiple cancer types, genomic analysis of brain metastasis has led to promising developments in novel therapies [31]. In this report, we identified several mutations present uniquely in the brain specimen, including those in the MAF, AURKB, FLG, HLA-C genes. We also identified an increase in SPEN RNA transcript expression in the same sample, which is a tumor repressor related to cell migration. However, the significance of these findings is unknown given the paucity of SMARCB1 cases.

In other rhabdoid tumors, such as atypical teratoid/rhabdoid tumors (AT/RT) of the CNS, loss of INI1 is associated with overexpression of cell-cycle regulatory protein cyclin D1, leading to cell cycle progression and untethered cellular proliferation [19, 20]. Interestingly, in a case series of 13 patients with SDSC by Kakkar et al., none of the SDSC tumors had significant immunoexpression of cyclin D1 [10]. This difference is a result of promoter methylation of the RASSF1α gene demonstrated in SDSCs [21]. We also found the SNP rs2073498 on RASSF1 reported in other cancers [32], expressed in the RNA of both sinonasal and brain specimens. These unique tumor characteristics and differences from other rhabdoid tumors further highlight the need for more epigenetic studies.

Histopathological diagnosis of this tumor can be complex as a number of malignant neoplasms can show similar histologic features and rhabdoid morphology in this location and should be considered in the differential diagnosis. ATRT has been reported in the sellar region of adults but these tumors were confined to the intracranial compartment and did not appear to predominantly involve the skull base and sinonasal regions [33]. Given the close association of the patient’s neoplasm with the cranial nerves, an epithelioid malignant peripheral nerve sheath tumor (eMPNST) should be considered, as about 2/3 of eMPNSTs can show INI-1 loss [34]. The absence of epithelial marker expression in eMPNST eliminated this as the diagnosis. NUT midline carcinomas of the sinonasal tract are aggressive neoplasms characterized by translocation of the NUT gene on chromosome 15q14 [3], but not INI-1 loss, an important consideration in the differential diagnosis for SMARCB1 (INI-1)-deficient sinonasal carcinomas. Likewise, other neoplasms with rhabdoid features do not show the INI-1 loss [35]. A number of neuroepithelial neoplasms have been identified to develop an AT/RT-like morphology, complete with SMARCB1 loss, however, this is an extremely rare occurrence [36]. With the advent of routinely using molecular markers to augment histopathological analysis, diagnosis of these rhabdoid subtypes is less challenging.

The progression of this patient’s tumor to leptomeningeal spread is an extremely rare occurrence. For cutaneous carcinomas, perineural invasion progressing to leptomeningeal spread has been previously reported [37]. However, to our knowledge this is the first report of a SMARC-B1 deficient tumor progressing to leptomeningeal spread. We advocate for other clinicians to continue to routinely sequence pathological tissue in order to gain understanding into the genetic drivers of metastasis for rare solid tumors.

## Additional files

Additional file 1: List of DNA mutations identified in each sample. (XLSX 22 kb)

Additional file 2: Quantitative Gene Expression differences between samples. (XLSX 10 kb)

Additional file 3: List of RNA mutational variants identified in each sample. (XLSX 13 kb)

## Data Availability

The datasets generated and analyzed during this current study are not publicly available, but are available from the corresponding author on reasonable request.

## Abbreviations

A: Adenine
ATRT: Atypical teratoid rhabdoid tumor
C: Cytosine
Ch: Chromosome
CT: Computed tomography
eMPNST: epithelioid malignant peripheral nerve sheath tumor
FLG: Filaggrin
G: Guanine
GKRS: Gamma knife radiosurgery
GRM3: Glutamate metabotropic receptor 3
hg 19: *Homo sapiens* (human) genome assembly GRCh37
IDH: Isocitrate dehydrogenase
Indel: Insertion and deletion of nucleotides
LOH: Loss of heterozygosity
MRI: Magnetic resonance imaging
SDSC: SMARCB1-deficient sinonasal carcinoma
SNP: single nucleotide polymorphism
T: Thymine
TCGA: The cancer genome atlas

## References

[1] Stelow Edward B., Jo Vicky Y., Mills Stacey E., Carlson Diane L. (2011). A Histologic and Immunohistochemical Study Describing the Diversity of Tumors Classified as Sinonasal High-grade Nonintestinal Adenocarcinomas. The American Journal of Surgical Pathology.

[2] Bishop JA. Recently described neoplasms of the sinonasal tract. Semin Diagn Pathol [Internet]. 2016 [cited 2018 Nov 27];33(2):62–70. Available from: http://www.ncbi.nlm.nih.gov/pubmed/26776744. 10.1053/j.semdp.2015.12.001 26776744

[3] Bishop JA, Westra WH. NUT midline carcinomas of the Sinonasal tract. Am J Surg Pathol [Internet]. 2012 [cited 2018 Nov 27];36(8):1216–1221. Available from: http://www.ncbi.nlm.nih.gov/pubmed/22534723. 10.1097/PAS.0b013e318254ce54 PMC4124635 22534723

[4] Stelow Edward B., Bellizzi Andrew M., Taneja Krishan, Mills Stacey E., LeGallo Robin D., Kutok Jeffery L., Aster Jon C., French Christopher A. (2008). NUT Rearrangement in Undifferentiated Carcinomas of the Upper Aerodigestive Tract. The American Journal of Surgical Pathology.

[5] Bishop Justin A., Guo Theresa W., Smith David F., Wang Hao, Ogawa Takenori, Pai Sara I., Westra William H. (2013). Human Papillomavirus-related Carcinomas of the Sinonasal Tract. The American Journal of Surgical Pathology.

[6] Bishop Justin A., Ogawa Takenori, Stelow Edward B., Moskaluk Christopher A., Koch Wayne M., Pai Sara I., Westra William H. (2013). Human Papillomavirus–related Carcinoma With Adenoid Cystic–like Features. The American Journal of Surgical Pathology.

[7] Bishop Justin A., Alaggio Rita, Zhang Lei, Seethala Raja R., Antonescu Cristina R. (2015). Adamantinoma-like Ewing Family Tumors of the Head and Neck. The American Journal of Surgical Pathology.

[8] Agaimy A, Koch M, Lell M, Semrau S, Dudek W, Wachter DL, et al. SMARCB1(INI1)-deficient sinonasal basaloid carcinoma: a novel member of the expanding family of SMARCB1-deficient neoplasms. Am J Surg Pathol [Internet]. 2014 Sep [cited 2018 Nov 27];38(9):1274–1281. Available from: http://content.wkhealth.com/linkback/openurl?sid=WKPTLP:landingpage&an=00000478-201409000-00014. 10.1097/PAS.0000000000000236 PMC4141899 24832165

[9] Agaimy Abbas, Hartmann Arndt, Antonescu Cristina R., Chiosea Simion I., El-Mofty Samir K., Geddert Helene, Iro Heinrich, Lewis James S., Märkl Bruno, Mills Stacey E., Riener Marc-Oliver, Robertson Thomas, Sandison Ann, Semrau Sabine, Simpson Roderick H.W., Stelow Edward, Westra William H., Bishop Justin A. (2017). SMARCB1 (INI-1)-deficient Sinonasal Carcinoma. The American Journal of Surgical Pathology.

[10] Kakkar A, Antony VM, Pramanik R, Sakthivel P, Singh CA, Jain D (2018). SMARCB1 (INI1) -deficient sinonasal carcinoma: a series of thirteen cases with assessment of histological patterns. Hum Pathol [Internet].

[11] Li H., Durbin R. (2009). Fast and accurate short read alignment with Burrows-Wheeler transform. Bioinformatics.

[12] Krassowski M, Paczkowska M, Cullion K, Huang T, Dzneladze I, Ouellette BFF, et al. ActiveDriverDB: human disease mutations and genome variation in post-translational modification sites of proteins. Nucleic Acids Res [Internet]. 2018 [cited 2019 Mar 10];46(D1):D901–D910. Available from: http://www.ncbi.nlm.nih.gov/pubmed/29126202. 10.1093/nar/gkx973 PMC5753267 29126202

[13] Krzywinski M., Schein J., Birol I., Connors J., Gascoyne R., Horsman D., Jones S. J., Marra M. A. (2009). Circos: An information aesthetic for comparative genomics. Genome Research.

[14] Dobin A, Davis CA, Schlesinger F, Drenkow J, Zaleski C, Jha S, et al. STAR: ultrafast universal RNA-seq aligner. Bioinformatics [Internet]. 2013 [cited 2019 Mar 10];29(1):15–21. Available from: https://academic.oup.com/bioinformatics/article-lookup/doi/10.1093/bioinformatics/bts635. 10.1093/bioinformatics/bts635 PMC3530905 23104886

[15] DePristo Mark A, Banks Eric, Poplin Ryan, Garimella Kiran V, Maguire Jared R, Hartl Christopher, Philippakis Anthony A, del Angel Guillermo, Rivas Manuel A, Hanna Matt, McKenna Aaron, Fennell Tim J, Kernytsky Andrew M, Sivachenko Andrey Y, Cibulskis Kristian, Gabriel Stacey B, Altshuler David, Daly Mark J (2011). A framework for variation discovery and genotyping using next-generation DNA sequencing data. Nature Genetics.

[16] Liao Yang, Smyth Gordon K., Shi Wei (2013). The Subread aligner: fast, accurate and scalable read mapping by seed-and-vote. Nucleic Acids Research.

[17] Dogan Snjezana, Chute Deborah J, Xu Bin, Ptashkin Ryan N, Chandramohan Raghu, Casanova-Murphy Jacklyn, Nafa Khedoudja, Bishop Justin A, Chiosea Simion I, Stelow Edward B, Ganly Ian, Pfister David G, Katabi Nora, Ghossein Ronald A, Berger Michael F (2017). Frequent IDH2 R172 mutations in undifferentiated and poorly-differentiated sinonasal carcinomas. The Journal of Pathology.

[18] Liu Xiaoling, Zhang Yu, Wang Zhiding, Wang Xiaoqian, Zhu Gaizhi, Han Gencheng, Chen Guojiang, Hou Chunmei, Wang Tianxiao, Shen Beifen, Li Yan, Ma Ning, Xiao He, Wang Renxi (2016). Metabotropic glutamate receptor 3 is involved in B-cell-related tumor apoptosis. International Journal of Oncology.

[19] Prickett Todd D, Wei Xiaomu, Cardenas-Navia Isabel, Teer Jamie K, Lin Jimmy C, Walia Vijay, Gartner Jared, Jiang Jiji, Cherukuri Praveen F, Molinolo Alfredo, Davies Michael A, Gershenwald Jeffrey E, Stemke-Hale Katherine, Rosenberg Steven A, Margulies Elliott H, Samuels Yardena (2011). Exon capture analysis of G protein-coupled receptors identifies activating mutations in GRM3 in melanoma. Nature Genetics.

[20] Dellovade T, Romer JT, Curran T, Rubin LL. The hedgehog pathway and neurological disorders. Annu Rev Neurosci [Internet]. 2006 [cited 2019 Mar 10];29(1):539–563. Available from: http://www.annualreviews.org/doi/10.1146/annurev.neuro.29.051605.112858 10.1146/annurev.neuro.29.051605.112858 16776596

[21] Wu Fujia, Zhang Yu, Sun Bo, McMahon Andrew P., Wang Yu (2017). Hedgehog Signaling: From Basic Biology to Cancer Therapy. Cell Chemical Biology.

[22] Kirschen GW, Xiong Q. Primary cilia as a novel horizon between neuron and environment. Neural Regen Res [Internet]. 2017 [cited 2019 Mar 10];12(8):1225–1230. Available from: http://www.nrronline.org/text.asp?2017/12/8/1225/213535. 10.4103/1673-5374.213535 PMC5607811 28966631

[23] Michaud EJ, Yoder BK. The primary cilium in cell signaling and cancer. Cancer Res [Internet]. 2006 Jul 1 [cited 2019 Mar 10];66(13):6463–6467. Available from: http://cancerres.aacrjournals.org/lookup/doi/10.1158/0008-5472.CAN-06-0462. 10.1158/0008-5472.CAN-06-0462 16818613

[24] Légaré S, Chabot C, Basik M. SPEN, a new player in primary cilia formation and cell migration in breast cancer. Breast Cancer Res [Internet]. 2017 Sep 6 [cited 2019 Mar 10];19(1):104. Available from: http://breast-cancer-research.biomedcentral.com/articles/10.1186/s13058-017-0897-3. 10.1186/s13058-017-0897-3 PMC5588740 28877752

[25] Jo Vickie Y, Chau Nicole G, Hornick Jason L, Krane Jeffrey F, Sholl Lynette M (2017). Recurrent IDH2 R172X mutations in sinonasal undifferentiated carcinoma. Modern Pathology.

[26] Roh J, Muelleman T, Tawfik O, Thomas SM. Perineural growth in head and neck squamous cell carcinoma: a review. Oral Oncol [Internet]. 2015 [cited 2019 Mar 24];51(1):16–23. Available from: http://www.ncbi.nlm.nih.gov/pubmed/25456006. 10.1016/j.oraloncology.2014.10.004 PMC4268058 25456006

[27] Chamberlain MC. Leptomeningeal metastasis. Curr Opin Oncol [Internet]. 2010 Nov [cited 2019 Mar 24];22(6):627–635. Available from: http://www.ncbi.nlm.nih.gov/pubmed/20689429. 10.1097/CCO.0b013e32833de986 20689429

[28] Dagan R, Bryant CM, Mendenhall WM, Amdur RJ, Morris CG, Lanza DC, et al. Isolated leptomeningeal progression from sinonasal carcinomas: implications for staging workup and treatment. Head Neck [Internet]. 2019 Mar 25 [cited 2019 Jun 16];hed.25741. Available from: http://www.ncbi.nlm.nih.gov/pubmed/30908735. 10.1002/hed.25741 30908735

[29] Kak M, Nanda R, Ramsdale EE, Lukas RV. Treatment of leptomeningeal carcinomatosis: current challenges and future opportunities. J Clin Neurosci [Internet]. 2015 Feb 9 [cited 2015 Feb 19]; Available from: http://www.ncbi.nlm.nih.gov/pubmed/25677875. 10.1016/j.jocn.2014.10.022 25677875

[30] Brastianos PK, Carter SL, Santagata S, Cahill DP, Taylor-Weiner A, Jones RT, et al. Genomic Characterization of Brain Metastases Reveals Branched Evolution and Potential Therapeutic Targets. Cancer Discov [Internet]. 2015 Nov [cited 2019 Mar 24];5(11):1164–1177. Available from: http://www.ncbi.nlm.nih.gov/pubmed/26410082. 10.1158/2159-8290.CD-15-0369 PMC4916970 26410082

[31] Venur VA, Cohen J V, Brastianos PK. Targeting Molecular Pathways in Intracranial Metastatic Disease. Front Oncol [Internet]. 2019 [cited 2019 Jun 16];9:99. Available from: http://www.ncbi.nlm.nih.gov/pubmed/30886831. 10.3389/fonc.2019.00099 PMC6409309 30886831

[32] Ferreira EN, Barros BDF, de Souza JE, Almeida RV, Torrezan GT, Garcia S, et al. A genomic case study of desmoplastic small round cell tumor: comprehensive analysis reveals insights into potential therapeutic targets and development of a monitoring tool for a rare and aggressive disease. Hum Genomics [Internet]. 2016 [cited 2019 Apr 6];10(1):36. Available from: http://www.ncbi.nlm.nih.gov/pubmed/27863505. 10.1186/s40246-016-0092-0 PMC5116179 27863505

[33] Paolini MA, Kipp BR, Sukov WR, Jenkins SM, Barr Fritcher EG, Aranda D, et al. Sellar region atypical Teratoid/Rhabdoid tumors in adults: Clinicopathological characterization of five cases and review of the literature. J Neuropathol Exp Neurol [Internet]. 2018 [cited 2019 Apr 7];77(12):1115–1121. Available from: https://academic.oup.com/jnen/article/77/12/1115/5116242. 10.1093/jnen/nly091 30295777

[34] Jo VY, Fletcher CDM. Epithelioid malignant peripheral nerve sheath tumor: clinicopathologic analysis of 63 cases. Am J Surg Pathol [Internet]. 2015 May [cited 2019 Apr 7];39(5):673–682. Available from: http://content.wkhealth.com/linkback/openurl?sid=WKPTLP:landingpage&an=00000478-201505000-00012. 10.1097/PAS.0000000000000379 25602794

[35] Perry A, Fuller CE, Judkins AR, Dehner LP, Biegel JA. INI1 expression is retained in composite rhabdoid tumors, including rhabdoid meningiomas. Mod Pathol [Internet]. 2005 Jul 11 [cited 2019 Apr 7];18(7):951–958. Available from: http://www.nature.com/articles/3800375. 10.1038/modpathol.3800375 15761491

[36] Schweizer Y, Meszaros Z, Jones DTW, Koelsche C, Boudalil M, Fiesel P, et al. Molecular transition of an adult low-grade brain tumor to an atypical Teratoid/Rhabdoid tumor over a time-course of 14 years. J Neuropathol Exp Neurol [Internet]. 2017 [cited 2019 Apr 7];76(8):655–664. Available from: https://academic.oup.com/jnen/article-lookup/doi/10.1093/jnen/nlx044. 10.1093/jnen/nlx044 28789476

[37] Dunn M, Morgan MB. Perineural invasion progressing to leptomeningeal carcinomatosis: is the absence of peripheral nerves an important sign? J Am Acad Dermatol [Internet]. 2010 [cited 2019 Mar 24];62(2):270–276. Available from: https://www.sciencedirect.com/science/article/pii/S0190962209009396. 10.1016/j.jaad.2009.06.083 20115949

